# Thermophilic biocatalysts for one-step conversion of citrus waste into lactic acid

**DOI:** 10.1007/s00253-023-12904-7

**Published:** 2024-01-20

**Authors:** Martina Aulitto, Alberto Alfano, Emanuela Maresca, Roberto Avolio, Maria Emanuela Errico, Gennaro Gentile, Flora Cozzolino, Maria Monti, Annachiara Pirozzi, Francesco Donsì, Donatella Cimini, Chiara Schiraldi, Patrizia Contursi

**Affiliations:** 1 Department of Biology, University of Naples “Federico II,”, Naples, Italy; 2 Institute for Polymers, Composites and Biomaterials (IPCB), National Research Council of Italy (CNR), Via Campi Flegrei 34, 80078 Pozzuoli, Italy; 3 Biological Systems and Engineering Division, Lawrence Berkeley National Laboratory, Berkeley, CA 94720 USA; 4 Department of Experimental Medicine, Section of Biotechnology, Medical Histology and Molecular Biology Naples, University of Campania L. Vanvitelli, Naples, Italy; 5 Department of Chemical Sciences, University of Naples “Federico II,” Naples, Italy; CEINGE Advanced Biotechnologies, Naples, Italy; 6 Department of Industrial Engineering, University of Salerno, Via Giovanni Paolo II 132, 84084 Fisciano, Italy; 7 NBFC, National Biodiversity Future Center, 90133 Palermo, Italy

**Keywords:** *W. coagulans*, Citrus waste, Fed-batch fermentation, Lactic acid

## Abstract

**Abstract:**

Agri-food residues offer significant potential as a raw material for the production of L-lactic acid through microbial fermentation. *Weizmannia coagulans*, previously known as *Bacillus coagulans*, is a spore-forming, lactic acid-producing, gram-positive, with known probiotic and prebiotic properties. This study aimed to evaluate the feasibility of utilizing untreated citrus waste as a sustainable feedstock for the production of L-lactic acid in a one-step process, by using the strain *W. coagulans* MA-13. By employing a thermophilic enzymatic cocktail (Cellic CTec2) in conjunction with the hydrolytic capabilities of MA-13, biomass degradation was enhanced by up to 62%. Moreover, batch and fed-batch fermentation experiments demonstrated the complete fermentation of glucose into L-lactic acid, achieving a concentration of up to 44.8 g/L. These results point to MA-13 as a microbial cell factory for one-step production of L-lactic acid, by combining cost-effective saccharification with MA-13 fermentative performance, on agri-food wastes. Moreover, the potential of this approach for sustainable valorization of agricultural waste streams is successfully proven.

**Key points:**

*• Valorization of citrus waste, an abundant residue in Mediterranean countries.*

*• Sustainable production of the L-( +)-lactic acid in one-step process.*

*• Enzymatic pretreatment is a valuable alternative to the use of chemical.*

**Graphical Abstract:**

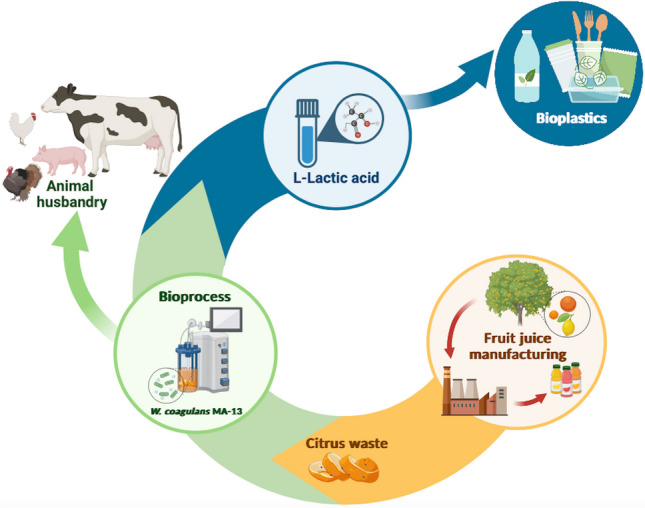

**Supplementary Information:**

The online version contains supplementary material available at 10.1007/s00253-023-12904-7.

## Introduction

The utilization of low-cost and renewable biomass as a source for producing valuable compounds is a major strategy to replace the petroleum-based materials (Sharma et al. 2017; Ahmad et al. 2020; Bradu et al. 2022). The use of biocatalysts, including microbes and enzymes, to develop green processes for the valorization of agri-food residues, is a hot topic of research in sustainable and zero-waste global development (Strazzulli et al. 2017; Ng et al. 2020; Tom et al. 2022). Recent examples of fermentation-based valorization strategies include anaerobic digestion of agri-food by-products, most of which employ lactic acid bacteria (LAB) as probiotics to enhance the nutritional value of several food matrices for functional food formulation (Ahmad et al. 2020). Additionally, LAB’s facultative anaerobic metabolism can be harnessed for the eco-sustainable production of lactic acid (LA), which serves as a building block for biodegradable bioplastics and finds applications in various biotechnological sectors, such as pharmaceutical, chemical, cosmetic, and food industries (Hofvendahl and Hahn-Hägerdal 2000; Ahmad et al. 2020; Ng et al. 2020). Microbial fermentation accounts for approximately 90% of total LA production due to several advantages over chemical synthesis, including reduced environmental impact through use of raw material and lower processing temperature (Hofvendahl and Hahn-Hägerdal 2000).

Among LA producers from renewable sources, various strains of *Weizmannia coagulans* have been utilized (Yankov 2022). Notably, a novel strain called MA-13, isolated from canned beans manufacturing, has been shown to be capable of efficiently producing LA from wheat straw, in a simultaneous saccharification fermentation (SSF) configuration (Aulitto et al. 2017). MA-13 showed an exceptional resistance towards toxic compounds derived from the thermo-acidic treatment of lignocellulose (Aulitto et al. 2019a). Unlike many microbial strains that produce racemic mixtures of L- and D-LA, optically pure L-LA is desired for numerous biotechnological applications due to its suitability for commercial use and simplified downstream purification processes (Okano et al. 2010; López-Garzón and Straathof 2014). Remarkably, MA-13 was found to synthetize only one of the two optical isomers of LA (L-LA), but its fermentation capabilities on agri-food wastes or untreated complex matrices have not been tested yet (Aulitto et al. 2017, 2019b).

Citrus wastes and other food by-products have been successfully used as carbon sources in culture media for microbial fermentation (Espinosa-Pardo et al. 2017). The global citrus processing volume reaches approximately 31.2 million tons annually, posing a significant ecological challenge (Suri et al. 2022). Citrus fruit production in Europe is concentrated in Mediterranean regions, with Spain accounting for nearly 60% of the EU’s total production, followed by Italy (around 30%) and smaller contributions from Cyprus, Greece, and Portugal. Solid/semisolid residues consist of peels (comprising 50–55% of the total fruit mass), seeds (20–40% of the total fruit mass), pomace, and wastewater (Zema et al. 2018; Suri et al. 2022). Citrus fruits are rich sources of vitamin C, folic acid, potassium, pectin as well as other phytochemicals and bioactive components, which offer health-promoting and therapeutic advantages, including antioxidant, anti-inflammatory, and anticancer properties (Zema et al. 2018; Suri et al. 2022). Some of these components find applications in food industries and other biotechnological sectors for the production of bioadsorbents, biofuels, biofertilizers, packaging material, activated carbon, and even incorporation into cosmetic products to provide unique fragrance through essential oils (Sharma et al. 2017; Pinto et al. 2021). Existing methods for valorizing citrus waste through anaerobic fermentation often require chemical pretreatments of the biomass, which have a high environmental impact (Wilkins et al. 2007). Consequently, there is an urgent need for eco-friendly and sustainable strategies/processes to alleviate the burden of waste materials (Cho et al. 2020). In this study, the use of MA-13 as microbial cell factory was investigated to establish an eco-sustainable process for LA production using raw citrus waste biomass, without any chemical pretreatment. The high conversion yield achieved highlights the potential of this microbe for fermentation-based valorization strategies.

## Material and methods

### Reagents

NaCl and tryptone were purchased from Biofroxx (Heidelberg, Germany). Yeast extract, glucose, and agar were supplied from Panreac AppliChem (Barcelona, Spain). M9 salts and carboxymethylcellulose were provided from Sigma-Aldrich (Darmstadt, Germany). Ortho-nitrophenyl-β-D-galactopyranoside (ONP-β-gal) and para-nitrophenyl-α-D-galactopyranoside (PNP-α-gal) used for testing the enzymatic activities were purchased from Biosynth (Staad, Switzerland). Cellic C-Tec2 (a commercial cocktail consisting of a blend of cellulases, β-glucosidases, and hemicellulases) was kindly provided from Novozymes A/S (Bagsværd, Denmark). The kit for L-lactic acid detection (L-lactic acid, L-lactate) assay kit (K-LATE) and xylan beechwood, wheat arabinoxylan, and glucomannan (konjac) were purchased from Megazyme (Bray, Irland). The citrus waste which is a by-product of fruit juice manufacturing was gifted from Ortogel SpA, Sicily, Italy.

### Proximate analysis and chemical composition of citrus waste

The chemical composition of citrus waste was determined by applying analytical methods. Moisture and ash content were evaluated by drying the waste at 105 °C in an forced-air oven (950.4 AOAC) and at 525 °C in a muffle (923.02 AOAC), respectively (Horwitz and Latimer 2005). The content of extractives, cellulose, hemicelluloses and lignin was determined using the gravimetric method according to the Technical Association of Pulp and Paper Industry (TAPPI) methods according to Pirozzi et al. (2022). Briefly, the extractives content was determined through Soxhlet extraction to remove sugars, phenolic compounds, and part of water-soluble polysaccharides, according to TAPPI (T-264 om-82) and ASTM E1721-01 methods (Pirozzi et al. 2023). Lignin extraction was performed according to the TAPPI T-222 om-22 method through acid hydrolysis on the extractive-free sample. Finally, cellulose isolation, based on the TAPPI T-203 method, was obtained through alkaline treatment with soda and acetic acid applied on extractive-free samples from the first isolation step. Pectin extraction and purification was performed according to Zannini et al. (2021).

### Enzymatic hydrolysis tests on citrus waste

Citrus waste was mechanically ground with a batch mill (IKA A11 basic, Staufen, Germany). Hydrolysis tests were conducted in 50-mL tubes on raw (< 25 mm) and ground (< 0.5 mm) citrus waste (peels, seeds, etc.) at different concentrations. The biomass was resuspended in a 5 mM sodium acetate buffer (pH 5.2, volume 40 mL) and sterilized in autoclave (Vapormatic 770, Napoli, Italy). Subsequently, Cellic CTec2 enzyme mixture (0.5 mL) was added to the mixture under sterile conditions. The hydrolysis was performed at 55 °C at 100 rpm in a shaking incubator (Lab Companion VWR, Milano, Italy) for 24 h. Subsequently, samples were centrifuged at 10,000 rpm (AvantiJ-20 XP, Beckman Coulter, Fullerton, CA, USA) to spin down the residual undigested biomass. The supernatant (1 mL) was ultrafiltered with 3 kDa membrane (Centricon, Amicon, Merck Millipore, Milano, Italy) before assessing the glucose concentration of the permeates via high-performance liquid chromatography (HPLC) (see “Analytical methods” section).

### Small-scale growth of *W. coagulans *MA-13 on citrus waste

*W. coagulans* MA-13, the bacterial strain used in this study, is deposited in Algal Collection at the University Federico II (ACUF) with the number 1–2023 (D’Elia et al. 2018). Cells were collected by centrifugation at 2600 × g for 10 min, and the pellets (25 OD_600nm_) were inoculated in 50 mL minimal medium (0.1% (w/v) yeast, M9 salts 1x, 1% (w/v) biomass) under the following conditions: (i) only with Cellic CTec2 enzymes (160 μL); (ii) only with MA-13 cells; and (iii) with a combination of the enzyme cocktails and MA-13 cells. The pH was adjusted to 5.5 with 2 M HCl. The 250-mL bottles equipped with hose nozzles were sparged with N_2_ for 3 min. The growth was carried out for 24 h at 55 °C and 180 rpm in an Innova shaking incubator (Innova 42, Eppendorf, Hamburg, Germany). The pH, cell viability (LB agar plates 1.8% (w/v)), and LA production of *W. coagulans* were evaluated after 0, 3, 6, 21, and 24 h. The survival ratio of MA-13 was determined by counting the number of viable cells on LB agar plates as colony-forming unit (CFU). CFU was calculated by dividing the number of colonies by the dilution factor and volume plated. The dry weight of the undigested citrus waste was measured at the end of cultivation. Residual biomass was filtered through the Miracloth membrane (EMD Millipore Corp., Burlington, MA, USA) and incubated at 60 °C overnight. The dry weight was measured using an analytical balance (Ohaus, Pioner, Darmstadt, Germany), and the residual biomass weight was expressed as percentage of the untreated biomass (incubated under the same conditions without enzymes and/or MA-13 cells) used as reference (100%).

### NMR (nuclear magnetic resonance) analysis

Untreated and treated biomass samples were analyzed by means of solid-state NMR, acquiring ^13^C spectra in both cross polarization (CP) under magic angle spinning (MAS) conditions. Spectra were collected at 100.47 MHz on a Bruker Avance II 400 spectrometer (Bruker Biospin, Billerica, MA, USA) operating at a static field of 9.4 T (i.e., Tesla), equipped with a 4-mm MAS probe. Finely ground samples were packed into 4-mm zirconia rotors and sealed with Kel-F caps (Bruker Biospin, Billerica, MA, USA), setting the spinning speed at 8 kHz for all experiments. CP spectra were acquired with a 1H π/2 pulse width of 3.0 µs, a contact time of 1.5 ms, and a recycle delay of 4 s, collecting 10,000 scans. Spectral deconvolution was performed using the software package Grams/8.0AI, THERMO Electron Corporation (Thermo Fisher Scientific, Waltham, MA, USA). A mixed Gaussian–Lorentzian line shape was chosen.

### Analysis of GH (glycosyl hydrolase) activities

Secretome and cell extract samples were prepared from the culture containing only MA-13 cells (see paragraph above) and cultivated for 24 h. Samples were screened for enzymatic activities over a panel of synthetic (PNP-α-gal, 2,2′-azino-di-(3-ethylbenzthiazoline sulfonic acid (ABTS)) and polymeric substrates (xylan, arabinoxylan, konjac, carboxymethyl cellulose (CMC)) at 55 °C and pH 6.5 (polymeric substrates) and pH 5.5 (artificial substrates). Supernatants and crude extract were prepared according to Aulitto et al. (2021). Briefly, 30 μL of the supernatants and 20 μg of crude extract were tested on (i) PNP-alpha-gal following the procedure described in Aulitto et al. (2021); (ii) on arabino-xylan, xylan, konjac, and CMC according to the 3,5-dinitrosalicylic acid (DNS) method (Carbonaro et al. 2022); and (iii) on ABTS (Ing et al. 2021; Salzano et al. 2022). The reaction activities were measured spectrophotometrically through Synergy H4 Plate Reader (BioTek Instruments Inc., Winooski, VT, USA). The reaction activities were expressed in international units (supernatants in mU/mL; crude extract in U/mg).

### Protein identification through LC–MS/MS (liquid chromatography tandem mass spectrometry)

Six micrograms of the secretome from MA-13 cells were grown as described above, and a control sample without cells was fractionated on 10% bis-acrylamide SDS-PAGE. The gel was stained with Coomassie Brilliant Blue (Thermo Fisher Scientific, Waltham, MA, USA), and 20 slices were cut from each lane and in situ trypsin digested, as previously reported (Butturini et al. 2016; Aulitto et al. 2021). Peptide mixtures were re-suspended in 0.2% formic acid and analyzed by nanoLC-MS/MS on an Orbitrap Exploris-240 coupled to the nanoLC system Vanquish (Thermo Fisher Scientific, Waltham, MA, USA). Samples were fractionated onto a C18 capillary reverse-phase column (150 mm, 75 μm, 2 μm 100°A) (Thermo Fisher Scientific, Waltham, MA, USA), working at a flow rate of 250 nl/min. A linear gradient from 2 to 90% developed in 45 min of eluent B (0.2% formic acid in 95% acetonitrile) in A (0.2% formic acid and 2% acetonitrile in LC–MS grade water (Merck, Darmstadt, Germany)) was employed.

The MS/MS method was set up in a data-dependent acquisition (DDA) mode, with a full scan ranging from 375 to 1200 m/z, followed by fragmentation of the top twenty ions (MS/MS scan) selected on the basis of intensity and charge state (+ 2, + 3, and multi-charges), and applying a dynamic exclusion time of 40 s. Raw data obtained from nanoLC-MS/MS were analyzed with MaxQuant 1.5.2 integrated (Cox and Mann 2008) with the Andromeda search engine for protein identification in MA-13 database containing the predicted protein sequences, deposited in National Center for Biotechnology Information (accession number SMSP00000000) (Aulitto et al. 2019a; 2022). The parameters selected for protein identification were the following: minimum 2 peptides, at least 1 unique; 0.01 FDR (i.e., false discovery rate) was used, with a reverse database for decoy; retention time alignment and second peptide search functions were allowed; trypsin as the proteolytic enzyme; missed cleavages maximum value of 3, mass tolerance value of 10 ppm for precursor ions, and 0.6 Da for MS/MS fragments; Cys carbamidomethylation as fixed modifications; and pyroglutamate (peptide N-terminal Gln) and Met oxidation as variable modifications (Zanca et al. 2010).

### Biomass pretreatment

Citrus waste was pretreated with aqueous ammonia following the protocol described by Ventrone et al. (2020). Briefly, dry biomass (5%) was incubated at 70 °C for 22 h in 10% (v/v) NH_4_OH. After centrifugation, the biomass was extensively washed with physiological solution and resuspended in a sodium acetate buffer 5 mM at pH to 5.2.

### Batch and fed-batch processes in bioreactor

Batch processes were performed in triplicate using 5 and 10% (w/v) of biomass. MA-13 was grown in Biostat CT plus (Sartorius Stedim, Gottingen, Germany) with a working volume of 1.8 L (total volume of 3.2 L) at 55 °C, pH 5.5, and 300 rpm. Nitrogen was initially sparged to obtain a percentage of dissolved oxygen lower than 10%, and the pH was controlled by adding 3 M NaOH. The preculture was grown in shake flask using LB medium under optimal conditions (55 °C and 180 rpm) up to 0.5 OD. Then, 1 L of culture was centrifuged and resuspended in 50 mL 0.1% yeast extract and used to inoculate 1.8 L of culture medium. For separate hydrolysis and fermentation (SHF) experiments, either untreated or ammonium hydroxide-treated biomass (5%) were enzymatically hydrolyzed (Cellic CTec2, 20 mL) for 17 h prior to strain inoculation.

Simultaneous saccharification and fermentation experiments (SSF) were performed by incubating the waste untreated biomass (10%) with Cellic Ctec2 (30 mL) up to 4 h before the addition of preculture (16.5 mL/L). Fed-batch processes were performed in triplicate by adding the biomass in two steps, i.e., 10% Cellic Ctec2 treated (4 h) citrus biomass was added simultaneously with the preculture and the glucose was regularly monitored until the residual concentration decreased below 5 g/L. Next, an additional amount (5%) of biomass plus 15 mL of enzyme solution was added to increase the LA production. Samples (10 mL) were withdrawn during the course of the SHF and SSF experiments to analyze residual glucose and LA.

### Analytical methods

The evaluation of glucose and organic acid concentration was performed through UHPLC Dionex Ultimate 3000 + chromatograph (Thermo Fisher, Milano, Italy) equipped with a UV/Vis and RI (refractive index) detector. The analytes were identified with a refractive index and an UV detector at 210 nm after separation on a Rezex™ ROA H + (8%) column (Phenomenex, Milano, Italy) eluted with 0.1% sulfuric acid at a flow rate of 0.8 mL/min and an oven temperature of 40 °C. Standard calibration curves of each analytes were built within a concentration range 0.01–30 mg/mL and used as reference.

## Results

### Chemical characterization of citrus waste

The data obtained from proximate analysis and the chemical composition of citrus waste are presented in Table 1. Cellulose and hemicellulose, to be used as sources of fermentable sugars in further experiments, accounted for about 50% of the biomass composition.

**Table 1:** Chemical composition of citrus waste is reported as relative percentage on dry basis (except for moisture)

Component	Citrus waste (wt%)
Moisture content	8.81 ± 0.46
Ash content	1.64 ± 0.09
Extractives	11.94 ± 0.47
Cellulose	31.33 ± 1.46
Hemicellulose	17.61 ± 0.86
Lignin	19.60 ± 0.95
Pectin	9.07 ± 0.10

*wt%* weight percentage

### Optimization of enzymatic hydrolysis on citrus waste biomass

**Fig. 1:**
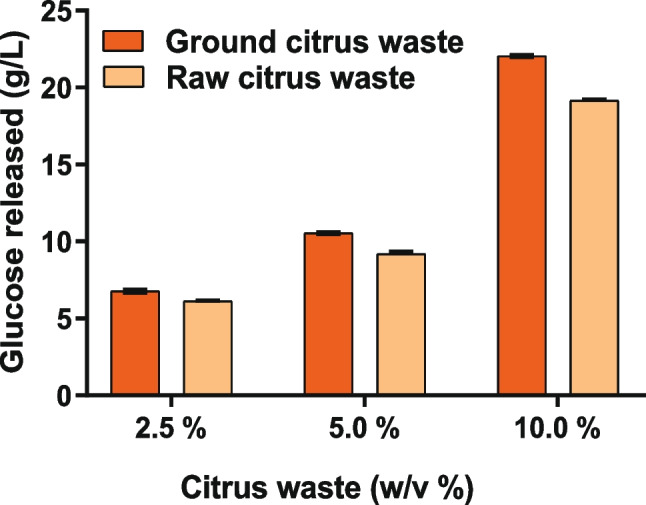
Test of Cellic Ctec2 hydrolysis efficiency on citrus waste. Different concentrations of untreated (raw citrus waste) and mechanically ground biomass were tested. Experiments were performed in duplicate

In this study, we aimed to establish a green and sustainable process for LA production, without employing any chemical pretreatments on the citrus biomass. Instead, we focused on enhancing the accessibility of hemicellulose and cellulose by mechanically grinding the biomass to reduce particle size (Silva et al. 2012). Untreated citrus waste (< 25.0 mm) and ground powdered citrus waste (< 1.0 mm) were used in small-scale hydrolysis experiments at three different concentrations (2.5, 5.0, and 10.0% w/v), and the release of glucose was analyzed by HPLC after enzymatic treatment with Cellic Ctec2 (Fig. 1). Surprisingly, we did not observe a significant improvement in enzymatic hydrolysis efficiency upon the grinding process. Only a 3.0% increase was revealed for the 10.0% (w/v) powdered sample, possibly due to the denser/viscous slurry obtained at higher citrus waste concentration, which enhanced mass transfer during hydrolysis. A similar, trend was observed for the 2.5% and 5.0% samples.

### Small-scale growth of *W. coagulans* MA-13 on citrus residues

#### Evaluation of biomass degradation

The evaluation of biomass degradation was conducted through preliminary screening tests in small scale (250-mL bottles) to determine the optimal conditions for SSF experiments. MA-13 was grown anaerobically on citrus residues in the presence and absence of Cellic Ctec2 cocktail.

**Fig. 2:**
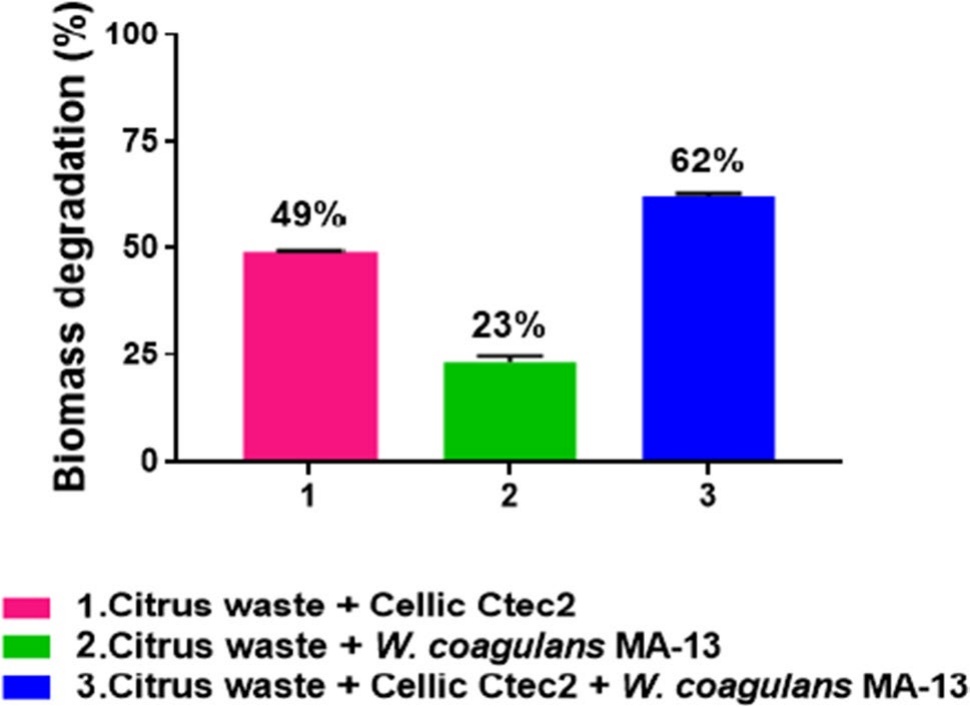
Effect of MA-13 and Cellic Ctec2 on citrus waste degradation after 24 h. Values are presented as mean values ± SD (*n* = 3)

**Fig. 3:**
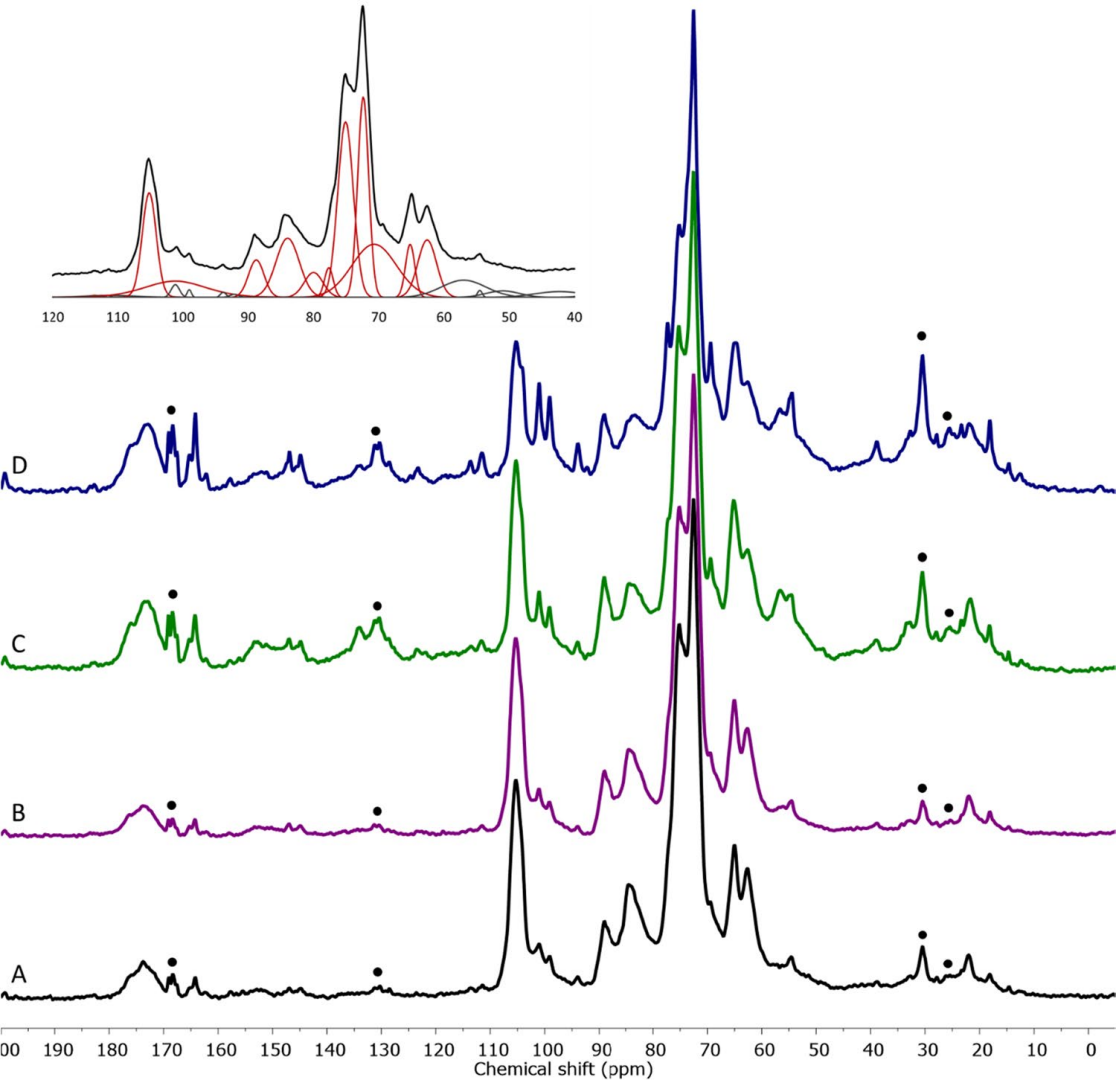
^13^C CP-MAS NMR spectra of citrus waste (A), citrus waste + MA-13 (B), citrus waste + Cellic Ctec2 (C) and citrus waste + Cellic Ctec2 + MA-13 (D). Peaks of liquid-like, low molecular weight substances, whose intensity is not reliably recorded in CP experiments, are marked by dots. In the insert, an example of deconvolution of the polysaccharide region is reported. NMR spectra were repeated on three independent biological samples

After 24 h, the dry weight reduction resulting from the degradative activity of MA-13 or Cellic Ctec2 was 23% and 49%, respectively (Fig. 2). Interestingly, when the cellular and enzymatic biocatalysts were combined, the percentage of citrus waste degradation increased to 62% (Fig. 2) demonstrating the synergistic effect of MA-13 and Cellic Ctec2 in improving the degradation yield. Figure 3 displays the ^13^C CP-MAS spectra of untreated and treated samples. Citrus waste consists of various polysaccharides (Table 1), which exhibit similar chemical moieties. The NMR spectra showed broad peaks due to the overlapping resonances of analogous chemical groups from different polysaccharides, making it challenging to assign specific signals. However, some spectral regions could be attributed to specific fractions of citrus waste. The polysaccharide signals and aromatics (including lignin) fell within the 50–110 ppm and 115–160 ppm range, respectively. Aliphatic resonances (10–40 ppm) indicated the presence of minor components, such as oils or waxes (extractives), while a broad and complex signal in the 170–180 ppm range corresponded to C = O groups of ester/carboxyl side chains in lignin and polysaccharides. A qualitative comparison of the spectra of biologically treated and untreated samples revealed an increased relative intensity of carbonyl/carboxyl, aromatic and aliphatic, and methoxy/ethoxy signals (50–60 ppm), primarily associated with pectins. This increase was particularly notable when the biomass was treated with Cellic Ctec2 alone or in combination with MA-13, suggesting that the biological treatment mainly affected the cellulose fraction. Semi-quantitative data obtained through spectral deconvolution support this hypothesis. Indeed, the ratio between the area of deconvoluted cellulose signals and the total signals in the spectra decreased in the following order: citrus waste (0.76) > citrus waste + MA-13 (0.74) > citrus waste + Ctec2 (0.57) > citrus waste + Ctec2 + MA-13 (0.53), consistent with the degradation trend shown in Fig. 2. Moreover, these NMR semi-quantitative data fit with the LA production as described below.

### Screening of intracellular and extracellular enzymatic activities

To identify the enzymatic activities involved in citrus waste degradation, intracellular cell extracts and the secretome of MA-13 were tested on a panel of natural polymeric (CMC, konjac, xylan, and arabinoxylan) and artificial (ABTS and PNP-α-Gal) substrates that mimic the chemical architecture of the main citrus waste polymers (Table 2).

**Table 2:** Enzymatic activities of MA-13 on a panel of natural and artificial substrates

	Enzymatic degradation activity	
Substrate	Intracellular (U/mg)	Secretome (mU/mL)
Xylan	n.d	n.d
Arabinoxylan	3.62 ± 0.085	n.d
Carboxymethylcellulose	4.28 ± 0.034	n.d
Konjac	3.83 ± 0.118	n.d
ABTS	n.d	n.d
PNP-α-Gal	n.d	17.03

Values are presented as mean values ± SD (*n* = 3)

Although the secretome analysis was hindered by low protein concentration, a relevant activity on PNP-α-Gal was revealed, indicating the presence of a GH capable of hydrolyzing α-1,6 linkages (Table 2). This finding is consistent with previous data showing that MA-13 secretes an extracellular α-galactosidase (WP_133537615.1) under standard growth conditions and on specific substrates, such as galactomannan substrates (locust bean gum) or complex agri-food matrices (i.e., rice hulls) (Aulitto et al. 2021). To shed further light on the enzymatic activities, the secretome was analyzed through LC–MS/MS. Sixty-nine proteins were identified from the MA-13 protein database (Supplemental Table S1), using the threshold of 5 peptides for each protein. Among them, 7% and 33% were linked to sugar degradation and carbohydrate metabolism, respectively. A single representative of GH3 family (WP_235962378.1) endowed with a typical Sec peptide as export signal was identified, whilst GH13 and GH65 enzymes (WP_133536578.1 and WP_133536961.1, respectively) lacked a canonical Tat or Sec secretion peptide (Aulitto et al. 2021). This is not surprising since it was previously demonstrated that MA-13 uses also alternative routes for protein exports (Aulitto et al. 2021). Most GH13 and GH65 members are generally active on starch α-1,4 and α-1,6 glycosidic bonds that might not be represented in citrus waste whose composition suggests the abundance of beta-glycosidic linkages (Supplemental Table S1). However, it must be underlined that GHs can exhibit a wider substrate specificity by exploiting ancillary activities as already demonstrated for a GH42 from MA-13 (Aulitto et al. 2021). Additionally, 59% of the identified extracellular proteins were associated with diverse functions of central metabolism. Conversely, the analysis of the intracellular enzymatic repertoire revealed the presence of hydrolyses capable of degrading almost all tested natural polymeric substrates, as shown in Table 2. The detected activity on arabinoxylan points to the auxiliary activity of intracellular β-galactosidase (WP_133536219.1) on such substrate. The presence of endoglucanases explained the observed activity on carboxymethylcellulose (Aulitto et al. 2017). Xylan degradation was not detected, likely due to the absence of xylan-degrading enzymes in the functional annotation of GHs in MA-13 genome. Finally, the only substrate for which no activity was detected intracellularly or extracellularly was ABTS, which is used to identify laccases. Although a putative gene encoding for a laccase is present in the MA-13 genome (MBF8419050.1), the lack of ABTS activity might be attributed to lack of specificity toward ABTS and/or inhibition mechanisms related to antioxidant molecules in citrus waste biomass and/or low gene expression levels (Ing et al. 2021). Overall, the previous data demonstrated that MA-13 is able to produce GHs when cultivated on citrus waste, although they represent a limited set of the GH repertoire available on MA-13 genome. Therefore, the degradation yield (23%, Fig. 2) achieved with cells alone can be explained by the concerted action of hydrolytic enzymes (Aulitto et al. 2018) along with the pH reduction, occurring during fermentation growth as a consequence of LA production (see below).

### Analysis of MA-13 growth parameters on citrus biomass

**Fig. 4:**
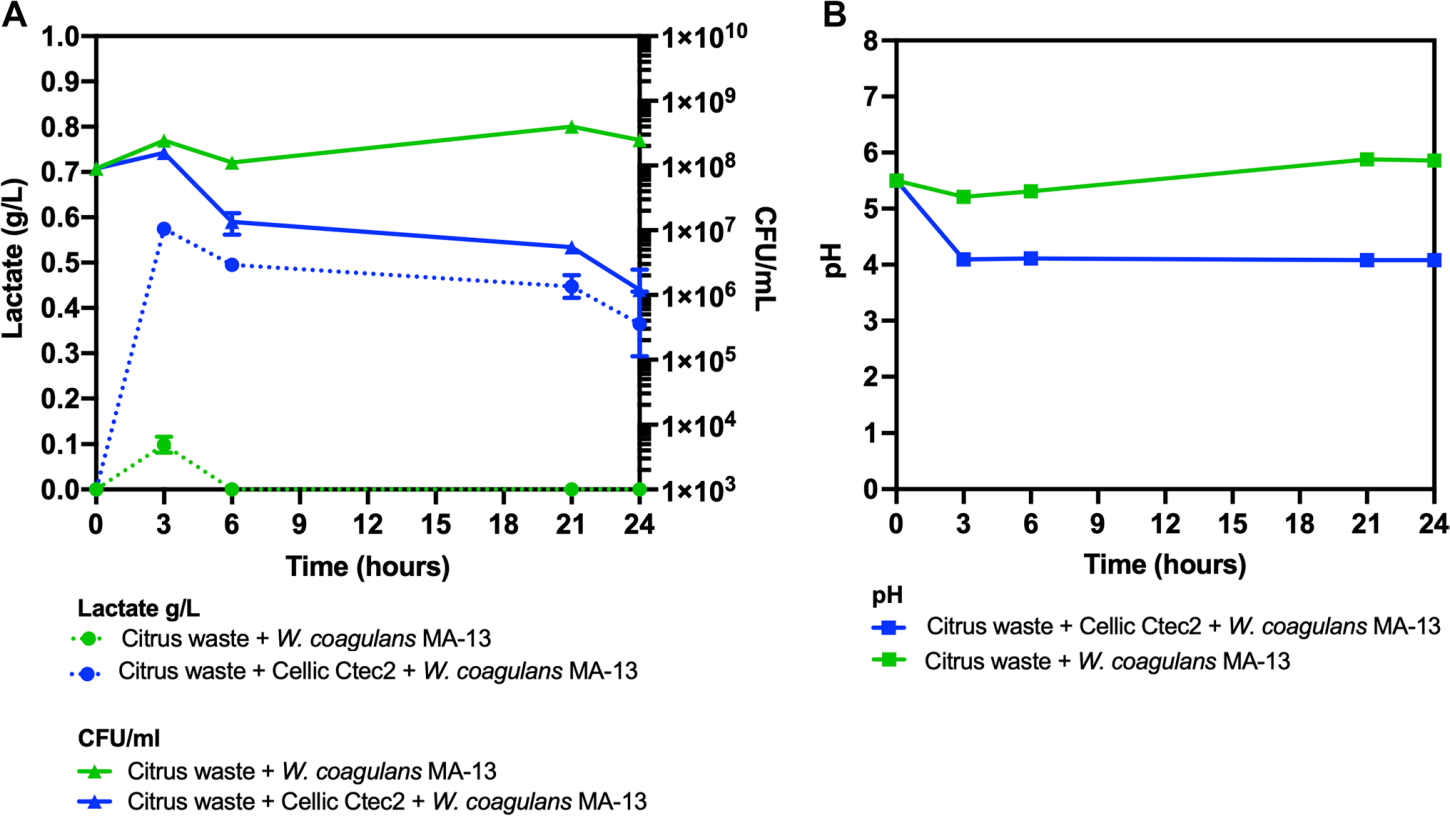
Growth parameters of MA-13 on citrus waste. **A** Lactate production (circle) and cell viability (CFU/mL) (triangle). **B** pH variation (in square). Values are presented as mean values ± SD (*n* = 3)

A small-scale growth experiment was used also to evaluate the ability of MA-13 to convert the citrus waste biomass into LA without any chemical pretreatment. Therefore, LA production was monitored thoroughly over the incubation, and it was found to peak at around 3 h, with the highest yield observed when Cellic CTec2 was added to the culture (~ 0.6 g/L vs ~ 0.1 g/L in the absence of the cocktail) (Fig. 4A).

Accordingly, the pH decrease, from 5.5 to 4.0, corresponded to increased LA production within the first 3 h and remained constant throughout the cultivation (Fig. 4B). Moreover, a decrease in cell viability (from ~ 10^8^ CFU/mL down to ~ 6 × 10^6^ CFU/mL) was observed, as indicated in the graph. Overall, these data suggest that the addition of Cellic CTec2 enhances LA production by boosting the release of fermentable sugars.

### Batch and fed-batch fermentation processes on 3 L bioreactors

Batch fermentations were carried out in 3 L bioreactors with two different concentrations of citrus waste (5 and 10%), under controlled pH and anaerobic conditions. In SSF configuration, untreated biomass was mixed with Cellic Ctec2 enzyme cocktail and growing cells. In the SHF configuration, the biomass was chemically pretreated prior to the saccharification step with Cellic CTec2 cocktail. As mentioned above, chemical or physical biomass processing is usually required to free cellulose and hemicellulose from the lignocellulosic matrix. Therefore, citrus waste was pretreated with ammonium hydroxide to evaluate its effect in releasing fermentable sugars upon biomass deconstruction. A comparative analysis of LA production on untreated and chemically treated biomass (at a 5% (w/v) load) was performed in SHF configuration. Results reported in Table 2 show that the pretreatment did not improve LA production (Table 2), demonstrating that the action of Cellic Ctec2 cocktail alone was effective in releasing fermentable sugars also from untreated citrus waste. Therefore, subsequent experiments were performed on untreated biomass. When 10% (w/v) citrus waste was used in SSF, the LA production increased from 16 g/L (SHF) to 28 g/L. Moreover, the productivity was also 76% higher (0.67 ± 0.10 g/L h vs 1.18 ± 0.11) in SSF compared to SHF configuration (Table 2). To further enhance LA production, the waste biomass load was increased to 15% in a fed-batch configuration. At this concentration, a dense and viscous slurry was observed, as consequence of citrus waste swelling in the aqueous medium (Piacenza et al. 2022) (Table 3).

**Table 3:** Comparison among parameters of different fermentation conditions. Standard growth parameters were volume, 1.8L; pH, 5.5; T, 55 °C; sparging of nitrogen, pO_2_ equal to 10%

Process type	Pretreatment with	Hydrolysis time and condition	Citrus waste (% w/v)	LA (g/L)	*Y* (p/s) (g_LA_/g_glu_)	Productivity Φ (gLA/Lh)
SHF batch*	10% (v/v) Ammonium hydroxide for 22 h	17 h, 1.6% (v/v) enzyme	5	16.0	0.97	0.67
SHF batch	-	17 h, 1.6% (v/v) enzyme	5	15.9 ± 1.1	0.97 ± 0.15	0.67 ± 0.10
SSF batch	-	4 h, 1.6% (v/v) enzyme	10	28.2 ± 2.0	0.94 ± 0.17	1.18 ± 0.11
SSF batch with pulse	-	4 h, 1.6% (v/v) enzyme	15 (10 + 5)	44.8 ± 0.3	0.96 ± 0.02	1.87 ± 0.01

*Single experiment

Values are presented as mean ± SD (*n* = 3)

**Fig. 5:**
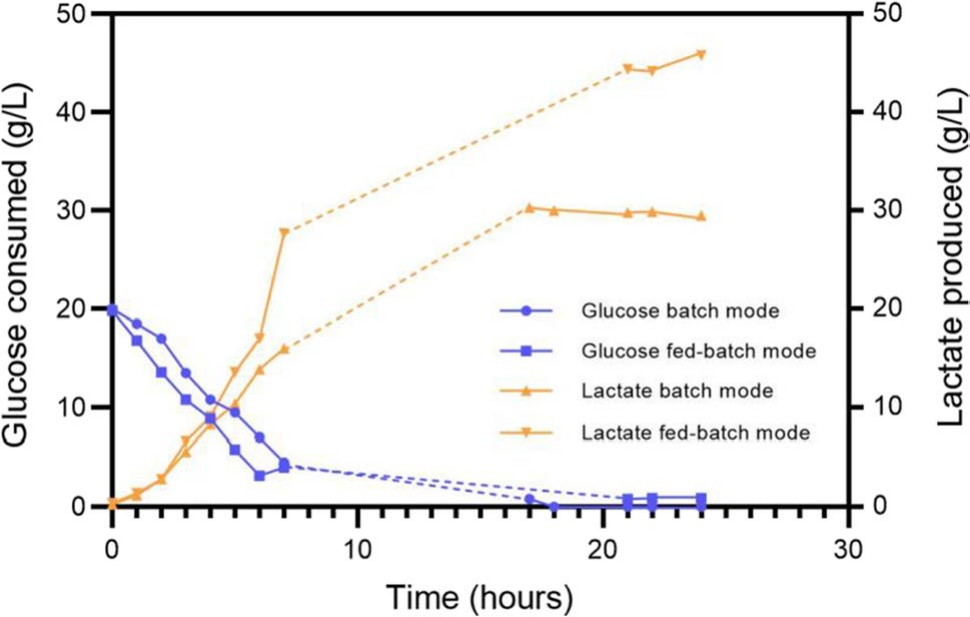
Time course of SSF batch and fed-batch experiments performed in triplicate with MA-13. Consumption of glucose and production of lactate in SSF are shown. A representative curve for each condition is shown in the graph

Therefore, MA-13 cells were first grown on 10% (w/v) biomass until the complete depletion of glucose occurred (6–7 h). Afterwards, the addition of another 5% (w/v) biomass aliquot increased LA production to about 44.8 g/L, with a productivity of 1.87 gLA/L/h, which was 60% higher than that obtained with 10% biomass. The results shown in Fig. 5 and Table 2 demonstrated the complete conversion of glucose into LA under all tested conditions and highlighted the increased productivity in fed-batch configuration of approximately 80% and 60% compared to those obtained in batch processes with 5% and 10% of biomass, respectively.

## Discussion

Notwithstanding the already existing biotechnological plants, the assessment and development of novel sustainable bioprocesses for production of added value molecules from agri-food and lignocellulose wastes are urgently required (Ahmad et al. 2020).

Annual production of LA is expected to reach 1960.1 × 10^3^ t in 2025, thus representing a challenging target in alternative to the petroleum-based materials. In this context, citrus wastes are attractive as a feedstock due to their abundance in fermentable C-5 and C-6 sugars contained mainly in cellulose and hemicellulose fractions (Zema et al. 2018; Suri et al. 2022; Mahato et al. 2021; Putri et al. 2022). The relative abundance of citrus waste components can be influenced by factors such as plant genetic variability, cultivation conditions, and harvest time (Ortiz-Sanchez et al. 2021). Comparing the data obtained in this study with the literature, we found that the content of cellulose, hemicellulose, and lignin (Table 1) aligns with the results obtained by Marin et al. (2007). Furthermore, it was demonstrated that soluble pentoses and hexoses in orange peel waste can account for up to 25–45% w/w (Rivas et al. 2008). Notably, the content of fermentable sugars present in the cellulose and hemicellulose fractions of the citrus waste used in this study is slightly higher (~ 48 wt%, i.e., weight percentage). Finally, the extractives, comprising approximatively 12 wt% of raw material, could serve as a rich source of bioactive compounds through conventional solvent extraction or innovative technologies like microwave, ultrasound, and high-pressure homogenization (Angiolillo et al. 2015; Boukroufa et al. 2015; Houfani et al. 2020; Wang et al. 2023).

The main objective of this work was to design a sustainable bioprocess by combining cost-effective saccharification of untreated biomass with the performance of MA-13 in LA production. Preliminary small-scale experiments evaluating performance of MA-13 on citrus waste (without chemical pretreatment) and LA production showed an increase of LA titers only after addition of the hydrolytic commercial enzymes. This result, however, was also accompanied by a decrease in cell viability (from ~ 10^8^ CFU/mL down to ~ 6 × 10^6^ CFU/mL), possibly due to pH variation and/or release of toxic compounds during enzymatic hydrolysis. On the other hand, the drop of cell viability was negligible compared to other microorganisms grown on similar substrates, indicating the robustness of MA-13 towards antimicrobial compounds present in citrus waste, such as limonene, terpenes, and phenolics (Bhatti et al. 2022). The finding is in line with the robustness toward lignocellulose biomass-derived inhibitors, as previously demonstrated (Aulitto et al. 2017; 2019b), indicating that MA-13 is a suitable microorganism to valorize also agri-food wastes for the production of valuable compounds.

The complex interaction networks among intertwined polymers within the cell wall often require harsh pretreatments to facilitate enzymatic breakdown (Hu and Ragauskas 2012; Preethi and Banu 2023). These processes are expensive and typically involve the treatment of effluents and/or solvent recovery (Alzahrani and Mohammad 2014). Conversely, in this study, a green physical grinding approach was attempted to improve the biomass dispersion in the fermentation broth (Sun et al. 2016) and its subsequent accessibility to enzyme attack. Results (Fig. 1) indicated that the reduction of citrus waste to fine particles only marginally facilitated enzyme action in breaking down the biomass’s polymeric structure. Nevertheless, we resolved to use the mechanical grinding to improve the citrus waste dispersion into the MA-13 growth medium. Small-scale analysis revealed that the degradation yield of MA-13 on such biomass was around 23%, and NMR data revealed that the decrease in dry weight involved mainly the cellulose fraction. Instead, the combination of the MA-13 GH repertoire along with Cellic CTec2 rose the degradation yield up to 62% demonstrating their synergistic effect on biomass deconstruction (Fig. 2).

Since small-scale growth lacks adequate regulation of crucial parameters, such as aeration and pH control (Aulitto et al. 2017), MA-13 was tested in SSF configuration. Previous studies demonstrated the feasibility of *W. coagulans* strains to ferment various agricultural and lignocellulose residues, such as corn stover, coffee, rice, straw, and bagasse residues, into LA, either alone or together with other bacteria and/or fungi, with yields ranging from 0.35 to 0.94 g/g (Yankov 2022; Ye et al. 2014; Neu et al. 2016; Ouyang et al. 2020; Cox and Mann 2008; Marzo-Gago and Venus 2022; Aulitto et al. 2017; 2019b). To the best of our knowledge, *W. coagulans* has not been tested on citrus waste up to date, therefore, we targeted MA-13 as a cell factory for one-step conversion into LA.

Orange peel wastes had been previously used as a feedstock for LA production through Lactobacilli sp., with conversion yields varying between 80 and 90% (Bustamante et al. 2019). The maximal yield of LA achieved in our study was 0.97 g_LA_/g_glu_, and under all the conditions tested, the glucose was completely fermented into LA pointing to MA-13 as a suitable microorganism for efficient biomass conversion. Fermentations with different process setups (SHF batch, SSF batch, SSF fed-batch) demonstrated optimal results in SSF conditions and an increase of the productivity of LA up to 1.87 g/L h in fed-batch processes. Interestingly, the yield and productivity were higher compared to other Lactobacilli strains (CECT 286 0.86 g/g, 0.63 g/L h; CECT 5037 0.84 g/g, 0.55 g/L h) (Bustamante et al. 2019). However, only ~ 30% of the total citrus waste was converted into LA in our study. It is known that MA-13 lacks at least some of the genetic determinants of the pentose phosphate pathway thus indicating that it only uses C-6 for conversion into LA (Aulitto et al. 2022).

Fermentation process parameters depend on sugar composition of the specific waste and/or the metabolic capability to convert C-5 (via the pentose phosphate or the phosphoketolase pathways) and C-6 (via the Embden–Meyerhof–Parnas pathway) (Wang et al. 2021). Further exploitation of MA-13 through genetic and/or metabolic strategies could enhance its fermentation potential, enabling it to utilize both C-6 and C-5 sugars.

Finally, the fermentation broth, once exhausted, can serve as a source of prebiotics after LA purification (Silvério et al. 2018; Fan et al. 2021). Indeed, MA-13 hydrolytic enzymes that are active on proteins and carbohydrates represent suitable prebiotic additives for improving the nutritional value of animal feed (Zhou et al. 2020).

Additionally, citrus waste has a rich composition of diverse bioactive compounds with antioxidant, anti-inflammatory, and anticancer properties (Rivas et al. 2008; Notomista et al. 2015; Espinosa-Pardo et al. 2017; Zema et al. 2018). The utilization of MA-13 as a microbial cell factory in bioprocesses based on agri-food wastes not only enables the production of bioactive compounds but also serves as an efficient bioconverter for lignocellulosic and agri-food residues. These findings point to MA-13 as an excellent model system for waste valorization, providing opportunities for sustainable utilization and the generation of value-added products.

## Supplementary Information

Below is the link to the electronic supplementary material.

Supplementary file1 (PDF 324 KB)

## Data Availability

The authors confirm that the data supporting the findings of this study are available within the article and/or its supplementary materials.
