# Supplementary material for: Thermophilic biocatalysts for one-step conversion of citrus waste into lactic acid
Source: Appl Microbiol Biotechnol. 2024 Jan 20;108:155. doi: 10.1007/s00253-023-12904-7 (PMC10799777; doi:10.1007/s00253-023-12904-7)
Supplement: Supplementary file 1 [file 253_2023_12904_MOESM1_ESM.pdf]

Supplementary materials

Applied Microbiology and Biotechnology

Thermophilic biocatalysts for one-step conversion of citrus waste into lactic acid

Martina Aulitto[1,3,4#], Alberto Alfano[2#], Emanuela Maresca[1], Roberto Avolio[3], Maria Emanuela Errico[3], Gennaro Gentile[3], Flora Cozzolino[5], Maria Monti[5], Annachiara Pirozzi[6], Francesco Donsì [6], Donatella Cimini[2*], Chiara Schiraldi [2] and Patrizia Contursi [1,7*]

[1]Department of Biology, University of Naples "Federico II", Naples, Italy

[2]Department of Experimental Medicine, Section of Biotechnology, Medical Histology and Molecular Biology Naples, University of Campania L. Vanvitelli, Naples, Italy

[3]Institute for Polymers, Composites and Biomaterials—IPCB, National Research Council of Italy (CNR), Via Campi Flegrei 34, 80078 Pozzuoli, Italy

[4]Biological Systems and Engineering Division, Lawrence Berkeley National Laboratory, Berkeley, CA 94720, USA

[5]Department of Chemical Sciences, University of Naples "Federico II", Naples, Italy; CEINGE Advanced Biotechnologies, Naples, Italy.

[6]Department of Industrial Engineering, University of Salerno, Via Giovanni Paolo II 132, 84084 Fisciano, Italy

[7] NBFC, National Biodiversity Future Center, Palermo 90133, Italy

[#]These authors equally contributed to the work

[*]Correspondence to: Patrizia Contursi (tel. 081679174, contursi @unina.it), Donatella Cimini (tel. 0815667686, donatella.cimini@unicampania.it)

TABLE S1: Proteins identified in the *W. coagulans* MA-13 secretome. Protein NCBI code, protein name, gel band in which the protein has been identified, protein molecular weight (kDa) and sequence coverage are reported.

Protein IDs	Protein name	Number of peptides	Gel band	Molecular weight [kDa]	Sequence coverage
WP_013859869.1	Thioredoxin	5	20	11,504	57.7
WP_041819467.1	MULTISPECIES: D-ribose pyranase	5	20	14,099	56.2
WP_019721883.1	DNA starvation/stationary phase protection protein	8	18	16,743	69.6
WP_017552418.1	Nucleoside-diphosphate kinase	7	17	16,599	47.3
WP_133536328.1	Manganese-dependent inorganic pyrophosphatase	16	10	33,786	63.8
WP_118499696.1	Aldo/keto reductase	11	10	34,386	39.3
WP_017550959.1	MULTISPECIES: ketol-acid reductoisomerase	10	10	37.72	42.1
WP_019720737.1	6-phosphofructokinase	13	9	34,255	54.7
WP_019721611.1	Asp-trna(Asn)/Glu-trna(Gln) amidotransferase subunit gatb	10	9	53,699	26.6
WP_195850273.1	Type I glyceraldehyde-3-phosphate dehydrogenase	21	8	36,257	71.6
WP_195850581.1	Malate dehydrogenase	15	8	33,468	69.5
WP_013858580.1	UDP-glucose 4-epimerase gale	9	8	37,142	32.6
WP_133536291.1	Porphobilinogen synthase	8	8	36,194	37.2
WP_035189134.1	Aldo/keto reductase	7	8	34,529	25.8
WP_014096744.1	MULTISPECIES: class II fructose-bisphosphatase	6	8	34,007	23.4
WP_195850412.1	Mannose-6-phosphate isomerase, class I	5	8	35,291	21.9
WP_133536554.1	Extracellular solute-binding protein	24	7	44,976	64.3
WP_133537323.1	Ornithine--oxo-acid transaminase	19	7	44,415	57.6
WP_029141825.1	Glycerol dehydrogenase	18	7	39,424	68.1
WP_014096089.1	MULTISPECIES: M42 family metallopeptidase	10	7	39,522	35.8

WP_029141503.1	Pyridoxal phosphate-dependent aminotransferase	10	7	42,969	37.5
WP_013858493.1	Lactonase family protein	9	7	37,557	40
WP_017553439.1	MULTISPECIES: NADH:flavin oxidoreductase/NADH oxidase	9	7	37,606	34.4
WP_195850272.1	Phosphoglycerate kinase	9	7	42,447	37.1
WP_013860504.1	MULTISPECIES: 2,3-butanediol dehydrogenase	8	7	37,467	24.9
WP_133536708.1	Molecular chaperone dnak	8	7	65,863	21.7
WP_133537094.1	Gfo/Idh/moca family oxidoreductase	8	7	37,189	36.3
WP_195850580.1	Citrate synthase	8	7	41,448	33.2
WP_118498790.1	Pyruvate dehydrogenase (acetyl-transferring) E1 component subunit alpha	7	7	41.29	28.3
WP_133536635.1	Iron-containing alcohol dehydrogenase	7	7	42,544	28.6
WP_017550325.1	MULTISPECIES: acyl-coa dehydrogenase	6	7	41,446	26.8
WP_013859914.1	NADP-dependent isocitrate dehydrogenase	24	6	46,796	49.8
WP_195850480.1	Phosphopentomutase	20	6	44,251	67
WP_195850181.1	Peptidase T	18	6	45,716	58.5
WP_234969572.1	Extracellular solute-binding protein	13	6	44,415	39.2
WP_017553805.1	Trigger factor	10	6	48,111	32.2
WP_013858135.1	Phosphoglucosamine mutase	6	6	48,345	18.4
WP_013860733.1	Serine hydroxymethyltransferase	6	6	45,371	18.4
WP_017552970.1	MULTISPECIES: adenylosuccinate lyase	5	6	49,686	14.4
WP_017553543.1	Xaa-Pro peptidase family protein	5	6	38,574	24.4
WP_195850403.1	Aspartate aminotransferase family protein	5	6	49,274	16.6
WP_195850260.1	NADP-dependent phosphogluconate dehydrogenase	17	5	52.31	54.3
WP_133537120.1	C1 family peptidase	16	5	51,388	43
WP_195850337.1	Aminopeptidase	16	5	45,354	54.3
WP_195850375.1	Type I glutamate--ammonia ligase	13	5	50,663	41.3
WP_017553024.1	Carboxypeptidase M32	12	5	58,403	33.8

WP_133535984.1	L-glutamate gamma-semialdehyde dehydrogenase	12	5	57,044	37.9
WP_118498902.1	Asp-trna(Asn)/Glu-trna(Gln) amidotransferase subunit gata	11	5	52,888	33
WP_195850489.1	Serine hydrolase domain-containing protein	10	5	60,055	22.9
WP_118498935.1	Peptide ABC transporter substrate-binding protein	9	5	60,812	24.6
WP_029142404.1	Acyl-coa dehydrogenase family protein	7	5	65,743	14.5
WP_133536243.1	Acetate--coa ligase	5	5	64,692	11.2
WP_133537615.1	Alpha-galactosidase	34	4	83,402	59
WP_029142773.1	2,3-bisphosphoglycerate-independent phosphoglycerate mutase	33	4	56,869	75.3
WP_133536562.1	Phospho-sugar mutase	33	4	64,615	62
WP_019721287.1	Formate C-acetyltransferase	29	4	85,419	55.7
WP_133536525.1	Oligoendopeptidase F	28	4	69,284	51.5
WP_133536923.1	Catalase	16	4	57,125	45.5
WP_029141784.1	M3 family oligoendopeptidase	14	4	69.12	33
WP_195850578.1	Pyruvate kinase	10	4	62,696	26.3
WP_235962378.1	Glycoside hydrolase family 3 protein	7	4	63,056	22.1
WP_017554503.1	Polyribonucleotide nucleotidyltransferase	6	4	77,718	11.6
WP_133536961.1	Alpha-amylase	6	4	56,026	14.4
WP_029142033.1	Malate synthase A	5	4	60.7	12.6
WP_133536578.1	Glycoside hydrolase family 65 protein	18	3	87,029	34.8
WP_133536909.1	3-hydroxyacyl-coa dehydrogenase/enoyl-coa hydratase family protein	16	3	87,377	24.9
WP_133536158.1	Glycoside hydrolase family 65 protein	12	3	88,156	20.1
WP_019720988.1	Alpha-glycosidase	5	3	69,995	11.1
WP_195850339.1	Aconitate hydratase acna	36	2	100.35	41.5
